# Correction: Analyzing and learning the language for different types of harassment

**DOI:** 10.1371/journal.pone.0232650

**Published:** 2020-04-29

**Authors:** The PLOS ONE Staff

## Notice of republication

This article was republished on April 16, 2020, to correct errors in the Introduction and Acknowledgments introduced during the typesetting process. The publisher apologizes for these errors. Please download this article again to view the correct version.
